# Editorial: Advances in crop breeding for abiotic stress tolerance

**DOI:** 10.3389/fgene.2024.1521062

**Published:** 2024-11-15

**Authors:** Yang Yang, Jianbo Li, Liang Chen

**Affiliations:** ^1^ Houji Laboratory of Shanxi Province, College of Agriculture, Shanxi Agricultural University, Taiyuan, Shanxi, China; ^2^ Plant Breeding Institute, School of Life and Environmental Sciences, The University of Sydney, Cobbitty, NSW, Australia; ^3^ State Key Laboratory of Crop Stress Biology for Arid Areas, College of Agronomy, Northwest A&F University, Yangling, Shaanxi, China

**Keywords:** cereal crop, abiotic stress, gene family, molecular and physiological responses, omics

## 1 Introduction

Global climate change, which includes drought, extreme temperatures, and adverse soil conditions such as salinity and heavy metal pollution, has had a profound impact on crop yields and quality, thereby posing a substantial threat to global food security (Waadt et al., 2022). To better adapt to a wide variety of abiotic stresses, cereal crops have undergone some fundamental changes in cellular processes and whole-plant physiology (Zhang et al., 2022). These adaptive responses are crucial for enhancing crop resistance and are of utmost significance for crop improvement (Gong et al., 2020). The identification of superior germplasm, the discovery of underlying mechanisms, and the utilization of important resistance genes are fundamentally important in the breeding of crops with abiotic stress resistance. Advanced techniques like high-throughput phenotype assessment, genome-wide association studies, multi-omics analysis, and gene editing have not only deepened our comprehension of the molecular mechanisms underlying crop responses to abiotic stresses but have also accelerated the breeding of crops with enhanced abiotic stress resistance (Gao, 2021).

Notwithstanding the fact that a multitude of strategies and significant genes involved in plants’ responses to abiotic stress have been reported in both model and non-model plants through the application of these advanced techniques, thereby augmenting our comprehension of the mechanisms underlying the abiotic stress tolerance of major crops, there still exist lacunae in knowledge. We instituted the Research Topic “*Advances in crop breeding for abiotic stress tolerance*” with the objective of bridging these gaps. This Research Topic encompasses the following themes: (a) Assessment of abiotic stress resistance and utilization of elite germplasm resources; (b) Identification of genes conferring abiotic stress resistance via genetic or genomic approaches, such as BSA-seq, QTL mapping, GWAS, and genome-wide characterization of crucial gene families; (c) Multi-omics investigations into the physiological and molecular mechanisms of crop abiotic stress tolerance/resistance; (d) Functional dissection of stress-related genes in crops by means of modern biotechnology; (e) Breeding strategies for abiotic stress tolerance in crops.

Within this Research Topic, we eventually accepted and published a total of seven articles, consisting of six Original Research and one Review. Altogether, 48 researchers were engaged in research pertaining to stress resistance in six species, namely, foxtail millet (*Setaria italica* L.), sorghum (*Sorghum bicolor* L.), common wheat (*Triticum aestivum* L.), quinoa (*Chenopodium quinoa* L.), common bean (*Phaseolus vulgaris* L.), and *Dendrobium huoshanense*. Ma et al. comprehensively reviewed the roles of hormone signal transduction, transcriptional factor regulation, and ROS system protective enzyme systems in the low-temperature response of wheat. Additionally, they presented a prospect on the future breeding strategies for enhancing cold resistance in wheat. The six original research papers will be elaborately dissected and interpreted in the following two parts.

## 2 Gene family involved in response to abiotic stress

- Zhou et al. systematically identified and characterized 70 members of the E3 ubiquitin ligase *U-box* gene family in foxtail millet and analyzed their expression patterns under salt stress. They confirmed that the *U-box* genes in foxtail millet (*SiPUB*) are relatively conserved and closely related to the homologs in rice. Meanwhile, they discovered several important candidate genes, such as *SiPUB20*, *SiPUB48*, and *SiPUB70*, that may be involved in the salt stress response of foxtail millet. This research has enhanced our understanding of the *U-box* gene family in foxtail millet and will contribute to the further application of *U-box* genes in the stress resistance breeding of foxtail millet.
- Gu et al. identified 22 DNA-binding with one finger (*Dof*) genes in *Dendrobium huoshanense.* Gene duplication and selection pressure analysis confirmed that the *DhDof* gene family did not undergo large-scale expansion and was under purifying selection. Eventually, based on qRT-PCR, they identified several *Dof* genes, such as *DhDof2* and *DhDof7*, that respond to hormones and cold stress. These results provided new insights for the further investigation of the *Dof* genes and the screening of the core stress-resistance genes.
- Guo et al. characterized six Drought-induced 19 (*Di19*) genes in common bean (*Phaseolus vulgaris* L.). Most of the *PvDi19*s were insensitive to salt stress and were induced by cold stress. *PvDi19-1* and *PvDi19-2* could rapidly respond to drought stress, and low-concentration Cd stress induced the upregulation of *PvDi19-6.* These findings enhance our understanding of the role of *PvDi19*s in the abiotic stress responses of common bean and provide a basis for future genetic enhancements in the stress tolerance of common bean.
- Mao et al. identified 49 *LLRLK* genes within sorghum and conducted a systematic analysis of their responses to diverse abiotic stresses. Via transgenic analysis, a crucial *LLRLK* gene (SORBI_3004G304700) was determined to be a negative regulator in relation to salt stress tolerance. Simultaneously, this gene was classified into three haplotypes, with Hap1 potentially being the predominant haplotype that mediates salt tolerance in sorghum. These results furnish valuable insights for comprehending the molecular mechanism by which *LLRLK* participates in the salt stress response of sorghum and for stress resistance breeding endeavors.
- Yang et al. identified 90 members of the *bZIP* gene family in foxtail millet and discovered that it had significantly expanded in foxtail millet due to segmental duplication. They also determined 12 *bZIP*s that were significantly upregulated under dehydration stress. This research has enhanced our understanding of the *bZIP* gene family in foxtail millet and provided candidates for the improvement of drought resistance and the analysis of the molecular mechanism underlying stress resistance in foxtail millet.

## 3 Molecular and physiological responses to abiotic stress

- Zhu et al. compared transcriptomic and physiological responses of drought-tolerant and susceptible genotypes of Quinoa. Drought-sensitive quinoa suffers from more oxidative damage, with reduced photosynthetic capacity and activated antioxidant enzymes. Multiple metabolic pathways such as the MAPK pathway, starch and sucrose metabolism, phenylpropanoid biosynthesis, and plant hormone signal transduction, as well as various transcription factors like AP2/ERF and MYB, are induced. This research not only helps us understand the drought resistance response of quinoa but also provides a comprehensive foundation for analyzing the drought tolerance molecular mechanism and breeding improvement of quinoa.

## 4 Prospect

Analyzing the responses of various crops to abiotic stress, especially identifying the relatively conserved metabolic pathways or important homologous genes in different species, is of crucial importance for the improvement of stress resistance breeding in important crops. In this Research Topic, the *U-box*, *Dof*, *Di19*, *bZIP*, and *LLRLK* gene families have been identified as potentially involved in the response process of crops to abiotic stress in different species. Multiple pathways related to transcriptional regulation, hormone regulation, energy metabolism, and secondary metabolite synthesis have also been found to be closely associated with abiotic stress. The interactions among these pathways/genes may be crosstalk, synergistic, or antagonistic, all of which have a certain impact on the abiotic stress resistance of crops. In the future, more advanced technologies and analytical strategies need to be further utilized to gain a deeper understanding of the molecular responses of crops to abiotic stress and ultimately promote the breeding improvement of crop stress resistance.
